# Programmed cell death 1 positive lymphocytes at palate tonsils in the elder patients with chronic tonsillitis

**DOI:** 10.1016/j.bbrep.2020.100898

**Published:** 2021-01-08

**Authors:** Yuki Fujihara, Koji Yamanegi, Yasuyuki Nagasawa, Ayu Yoshida, Yukako Goto, Shunsuke Kumanishi, Hiroyuki Futani, Shigeo Fukunishi, Shinichi Yoshiya, Hiroshi Nishiura

**Affiliations:** a Department of Orthopedic Surgery, Hyogo College of Medicine, Nishinomiya, Hyogo, 663-8501, Japan; b Department of Pathology, Hyogo College of Medicine, Nishinomiya, Hyogo, 663-8501, Japan; c Department of Internal Medicine, Division of Kidney and Dialysis, Hyogo College of Medicine, Nishinomiya, Hyogo, 663-8501, Japan; d Department of Otorhinolaryngology Ear Nose Throat, Konan Hospital, Kobe, Hyogo, 663-8501, Japan

**Keywords:** Chronic tonsillitis, GPR56, Lymphocytes, Marginal zone, PD-1, C5a receptor, (C5aR), cytotoxic T lymphocyte-associated protein-4, (CTLA-4), G protein-coupled receptor, (GPCR), Janus kinase 1, (JAK1), programmed cell death 1, (PD-1), ribosomal protein S19, (RP S19), signal transducers and activator of transcription 1, (STAT1), T cell receptor, (TCR), type I interferons, (IFNα and IFNβ)

## Abstract

Circulating lymphocytes infiltrate into local foci at the inflammatory phase of acute wound healing for activation of the immune system and express an immune checkpoint protein programmed cell death 1 (PD-1) at the resolution phase for inactivation of the immune system. Conversely, the PD-1 expression was still found even on circulating lymphocytes of the elder patients with chronic tonsillitis at the palliative stage. Recently, an adhesion G protein coupled receptor 56 (GPR56) was reported to at least work as a proliferation factor for infiltrated lymphocytes into local foci at the resolution phase of acute wound healing. To preliminary examine a similar role of PD-1 and GPR56 at local foci at chronic inflammation, palate tonsils were prepared from small amounts of patients with chronic tonsillitis and tonsillar hypertrophy. A positive relationship of RNA expression might be observed between PD-1 and GPR56 in the elder patients with chronic tonsillitis. In regard to immunohistopathological findings, there were huge and small amounts of PD-1 and GPR56 expression at the marginal zone of lymphoid follicles of palate tonsils with chronic tonsillitis. Moreover, the positive relationship of RNA expression between PD-1 and GPR56 confirmed in large numbers of the elder patients with chronic tonsillitis. Probably, GPR56 participates in a supplement of PD-1^+^ lymphocytes to circulating bloods of the elder patients with chronic tonsillitis through a lymphocyte cell maintenance system at the marginal zone of the lymphoid follicles of palate tonsils.

## Highlights

• Accumulation of PD-1^+^ lymphocytes at the marginal zone of palate tonsils.
• Expression of GPR56 transcript at palate tonsils.
• Co-localization of GPR56^+^ lymphocytes besides PD-1^+^ lymphocytes.
• Positive relationship between GPR56 and PD-1 in palate tonsils of chronic tonsillitis-patients.

## 1 Introduction

Acute inflammation is often divided into three phases to explain the wound healing process for a short period: the initiation, the inflammation, and the resolution [[1], [2], [3]]. Circulating lymphocytes are believed to infiltrate into local foci at the inflammation phase of acute wound healing for identifying foreign antigens on M1 macrophages activated by type II interferon (IFN gamma) in the mature immune system [4]. Type II IFN is produced in lymphocytes and natural killer cells for enhancement of innate immunity at the inflammation phase of acute wound healing. Conversely, infiltrated lymphocytes also express an immune checkpoint protein programmed cell death 1 (PD-1) for a short period through an induction of type I IFNs (IFN alpha and IFN beta) at the resolution phase of acute wound healing. Type I IFNs are produced in neutrophils and M2 macrophages for regulation of the mature immune system at the resolution phase of acute wound healing. Oppositely, PD-1 ligand 1/2 (PD-L1/L2) on M2 macrophages plays a key role in the limitation of excessive activation of the mature immune system at the resolution phase of acute wound healing [5].

The nature of circulating lymphocytes in patients with chronic tonsillitis was newly found to be different from that with a peritonsillar abscess as an acute severe tonsillitis or tonsil hyperplasia [6]. A state of immune exhaustion or senescence circulating lymphocytes were immunohistochemically shown by using anti-PD-1 antibodies in patients even at around 30 years with chronic tonsillitis. The PD-1-mediated signal interferes with the T cell receptor (TCR)-mediated signal for recognition of any kind of antigens [7]. Moreover, CD69 have been highly detected on PD-1^+^ circulating lymphocytes in patients with chronic tonsillitis. CD69 is a marker of resident memory lymphocytes at local foci for producing antibodies through the activation of B cells. It is likely that the acquired immune system at whole body suppressed by the PD-1-mediated signal decrease a chance of PD-1^-^/CD4^+^ lymphocyte-helped recognition to non-autoantigen on antigen presenting cells. The competitive inhibition is one of the causes at least for delaying acute inflammation. The mechanism for expanding PD-1^+^ lymphocytes at the local foci and/or the whole body needs to be examined.

A subset of effector memory lymphocytes in circulating bloods was reported to arrive at local foci with acute inflammation and re-expressed naive T cell marker CD45RA after antigenic stimulation [8]. CD45RA^+^ effector lymphocyte subsets expressed GPR56 and received a high potency of clonal expansion. This report suggested a role of GPR56 on infiltrated circulating lymphocytes into local foci in the proliferation of PD-1^+^ lymphocytes at the resolution phase of acute inflammation or the development phase of chronic inflammation. To preliminary examine the similar role of GPR56 in proliferation of PD-1^+^ lymphocytes at tonsil at chronic inflammation, small numbers of patients with tonsillar hypertrophy and chronic tonsillitis were prepared for this study.

## 2 Materials and methods

### 2.1 Patients

There were 9 patients with tonsillar hypertrophy and 13 patients with chronic tonsillitis in Konan Hospital. There were 39 patients with chronic tonsillitis in Meiwa Hospital. These study designs were approved by our institutional review board (No. 25) and informed consent was obtained from all patients. The clinical data before operation are shown in Table 1 The small fragments of the fresh palate tonsils after operation were put in RNA*later*™ and kept at −80 °C until RNA preparation. The remaining samples were fixed in 10% formalin for paraffin blocks.

### 2.2 Antibodies

Anti-mouse PD-1 rat IgG2a (clone: 29F.1A12), anti-human GPR56 rabbit IgGs or anti-human cytotoxic T lymphocyte-associated protein-4 (CTLA-4) mouse IgG were produced by Bio X Cell (Kyoto, Japan), Bioss (MA, USA) or Santa Cruz Biotechnology (CA, USA), respectively. HRP-conjugated anti-rat IgGs rabbit IgGs, anti-rabbit IgGs goat IgGs or anti-mouse IgGs sheep IgGs were purchased from Santa Cruz Biotechnology.

### 2.3 Immunohistochemistry

Paraffin sections of 4 μm thickness were stained with hematoxylin and eosin using typical methods. At the same time, paraffin sections were reacted first and HRP-conjugated secondary antibodies and DAB Substrate-Kit (Agilent Tec.) in histostainer36A system according to a standard manual for detection of specific proteins (Nichirei Corporation, Tokyo, Japan). Tissues were observed using an automatic microscope, BX50 and a digital camera, DP22 by CellSens Standard software (Olympus, Tokyo, Japan). The DBA signal was measured as the cell density by NIH ImageJ software (64-bit Java 1.8.0_112).

### 2.4 PCR

All RNA in organs were prepared by RNeasy Mini Kit (QIAGEN, Hilden, Germany). After confirming the quality of RNA by RNA 6000 Nano Kit (Agilent Tec., Tokyo, Japan), each cDNA was prepared by RT-PCR using the Takara PrimeScript™ RT reagent Kit according to the instruction manual. RT was performed under the following conditions: temperatures of the RT and the denature were 37 and 85 °C, respectively. The time periods were 15 min and 5 s, respectively.

The transcription levels were analyzed by semi-quantitate PCR using KOD One® PCR Master Mix (TOYOBO CO., LTD. Osaka, Japan) with specific primer pairs (PD-1 forward primer, 5′-CATCGGAGAGCTTCGTGCTA-3′ and PD-1 reverse primer, 5′-GTGCGCCTGGCTCCTATT-3’: GPR56 forward primer, 5′-AGCCAGTTCCTGAAGCATCC-3′ and GPR56 reverse primer, 5′-TTCTTCGGCTGTAGCTGGTG-3′ or beta-actin forward primer, 5′-ACAGAGCCTCGCCTTTGC-3’: beta-actin reverse primer, 5′-GCGGCGATATCATCATCC-3’: and GAPDH forward primer, 5′-CATGTTCGTCATGGGGTGAACCA-3′ and GAPDH reverse primer, 5′-AGTGATGGCATGGACTGTGGTCAT-3′) according to the instruction manual. PCR was performed under the following conditions: temperatures of the denature, the annealing, and the elongation were 94, 58, and 72 °C, respectively. The time periods were 10 s, 20 s and 30 s, respectively. The elongation cycle was 30 cycles. The level of transcription was measured using NIH ImageJ software according to the following formula: relative transcription rate of sample gene to beta-actin gene (density of sample band/density of beta-actin band).

### 2.5 Statistical analysis

The results of the representative examinations were confirmed by multiple experiments with at least triplicate samples. Statistical significance was calculated by either non-parametric or parametric tests in the two-way analysis of variance window. The values are expressed as the mean ± SD. A p-value <0.05 was considered statistically significant and is shown as P < 0.05: * and P < 0.01: **.

## 3 Results

### 3.1 Clinical data of patients with tonsillar hypertrophy and chronic tonsillitis

**Fig. 1:**
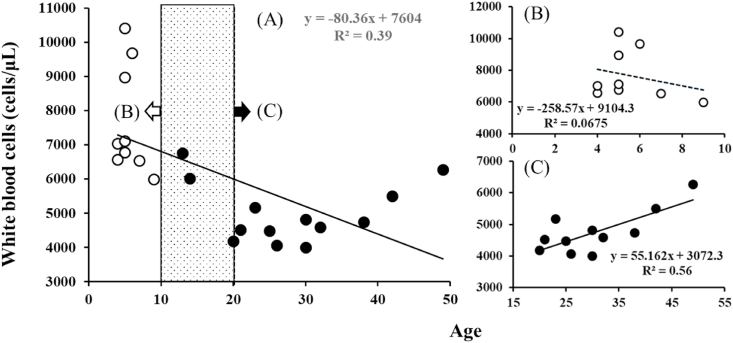
The age-dependent circulating white blood cell numbers of patients with tonsillar hypertrophy and chronic tonsillitis. The personal data about age and circulating white blood cell numbers of patients with tonsillar hypertrophy and chronic tonsillitis were plot, and the approximate straight line and R^2^ were automatically drawn by Microsoft Excel.

Patient data for the tonsillar hypertrophy group and chronic tonsillitis group were as follows: Number of patients: 9 and 13; age range; 5.6 ± 1.6 and 27.9 ± 10.5; female to male ratio: 5/4 and 5/8; white blood cell numbers (cells/μL) or C reacted proteins: 7667.8 ± 1581.8 and 5006.9 ± 880.3 or 0.05 ± 0.06 and 0.2 ± 0.3. Significant differences of age (P = 0.0000040) and white blood cell numbers (P = 0.000059) between the tonsillar hypertrophy and chronic tonsillitis groups were found (Table 1). However, there was an inverse relationship (Y = −80.36x + 7604, coefficient of determination (R^2^) = 0.39) between age and white blood cell numbers (Fig. 1A). To study the effects of the different immune system in an age dependent manner on development of acute and chronic tonsillitis, the relationship between age and white blood cell numbers was re-analyzed. There was an inverse relationship (Y = −258.57x + 9104.3, R^2^ = 0.0675) between age and white blood cell numbers in under 10 years old (young) patients (Fig. 1B). On the contrary, there was a positive relationship (Y = 55.162x + 3072.3, R^2^ = 0.56) between age and white blood cell numbers in above 20 years old (elder) patients (Fig. 1C). Age seems to become the best marker to distinguish chronic tonsillitis with tonsillar hypertrophy.

**Table 1:** Data for tonsillar hypertrophy patients and chronic tonsillitis patients.

patient	disease	sex	Age(year)	Gpr56/actin (ratio)	WBC (number/μL)	Neutrophils (%)	Lymphocyte (%)	Monocyte (%)	CRP
MH1	tonsillar hypertrophy	male	4	0.75	6560	62.2	26.2	5.3	0.04
MH2			5	0.74	8960	37.3	50.3	5.5	0.05
MH3			5	1.76	6770	52.2	35.9	3.4	0.03
MH4			6	0.88	9670	39.1	51.8	5.0	0.00
FH1		female	4	1.16	7020	42.5	48.2	3.5	0.01
FH2			5	0.95	10400	53.8	35.8	5.1	0.04
FH3			5	1.07	7110	53.2	35.5	3.8	0.06
FH4			7	1.28	6530	53.7	33.8	4.6	0.20
FH5			9	1.86	5990	44.5	41.6	4.1	0.04
	average		5.6	1.16	7667.8	48.7	39.9	4.5	0.05
	SD		1.6	0.41	1581.8	8.2	8.7	0.8	0.06

MC1	chronic tonsillitis	male	13	1.09	6750	43.6	42.9	3.1	0.00
MC2			14	1.54	6010	48.0	37.0	12.0	0.64
MC3			20	0.77	4180	49.0	42.0	7.0	0.95
MC4			23	0.87	5170	45.9	41.5	6.3	0.01
MC5			25	1.78	4480	52.8	36.7	5.6	0.01
MC6			30	1.31	4000	53.6	38.2	5.0	0.03
MC7			32	1.71	4590	57.8	35.3	3.9	0.05
MC8			42	1.43	5500	50.2	39.0	5.2	0.07
FC1		female	21	1.53	4520	39.9	48.1	5.2	0.02
FC 2			26	0.83	4060	47.1	41.4	7.5	0.28
FC 3			30	1.71	4820	53.0	39.0	4.0	0.03
FC 4			38	1.28	4740	67.7	25.3	3.5	0.33
FC 5			49	1.71	6270	51.1	35.3	7.6	0.01
	average		27.9	1.35	5006.9	50.7	38.6	5.8	0.19
	SD		10.5	0.36	880.3	6.9	5.3	2.4	0.30
H & C	two-ways		4.0E-06	0.27	5.9E-05	0.54	0.66	0.1	0.20

MH: male patient with tonsillar hypertrophy, FH: female patient with tonsillar hypertrophy, MC: male patient with chronic tonsillitis, FC: female patient with chronic tonsillitis.

**Fig. 2:**
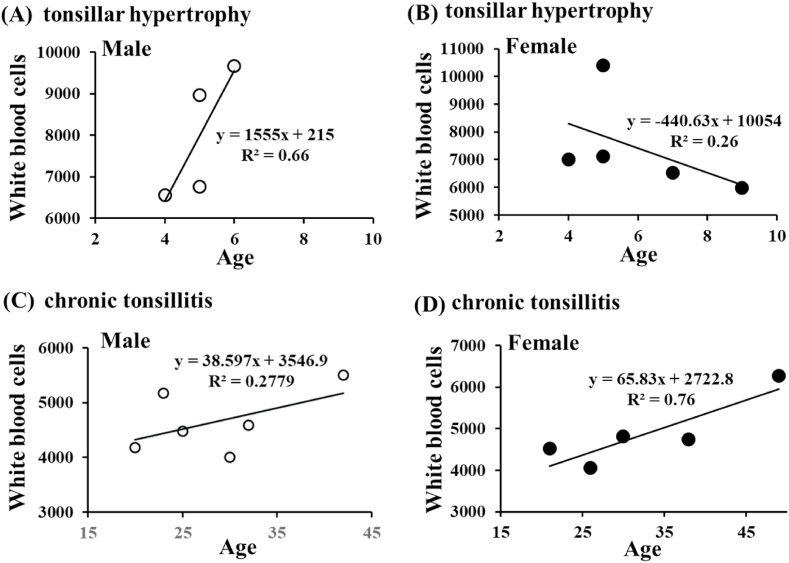
The age- and sex-dependent circulating white blood cell numbers of patients with tonsillar hypertrophy and chronic tonsillitis. The personal data about age and circulating white blood cell numbers of patients with tonsillar hypertrophy and chronic tonsillitis were separately plot by sex, and the approximate straight line and R^2^ were automatically drawn by Microsoft Excel.

To further study the effects of the sex-dependent immune system on development of chronic tonsillitis, patients with tonsillar hypertrophy and chronic tonsillitis were separated by sex and the relationship between age and white blood cell numbers was analyzed. There was a positive relationship (Y = 1555x + 215, R^2^ = 0.66) between age and white blood cell numbers of male patients with tonsillar hypertrophy (Fig. 2A). However, the age range of male patients with tonsillar hypertrophy was very narrow. On the contrary, there was an inverse relationship (Y = −440.63x + 10054, R^2^ = 0.26) between age and white blood cell numbers of female patients with tonsillar hypertrophy (Fig. 2B). Conversely, there was a positive relationship (Y = 38.597x + 3546.9, R^2^ = 0.2779) between age and white blood cell numbers of male patients with chronic tonsillitis (Fig. 2C), and there was a positive relationship (Y = 65.83x + 27722.8, R^2^ = 0.76) between age and white blood cell numbers of female patients with chronic tonsillitis (Fig. 2D). From small numbers of above clinical data, there was the age-dependent expansion of white blood cells in the elder patients with chronic tonsillitis.

### 3.2 The participation of Gpr56 in the elder immune system of chronic tonsillitis

**Fig. 3:**
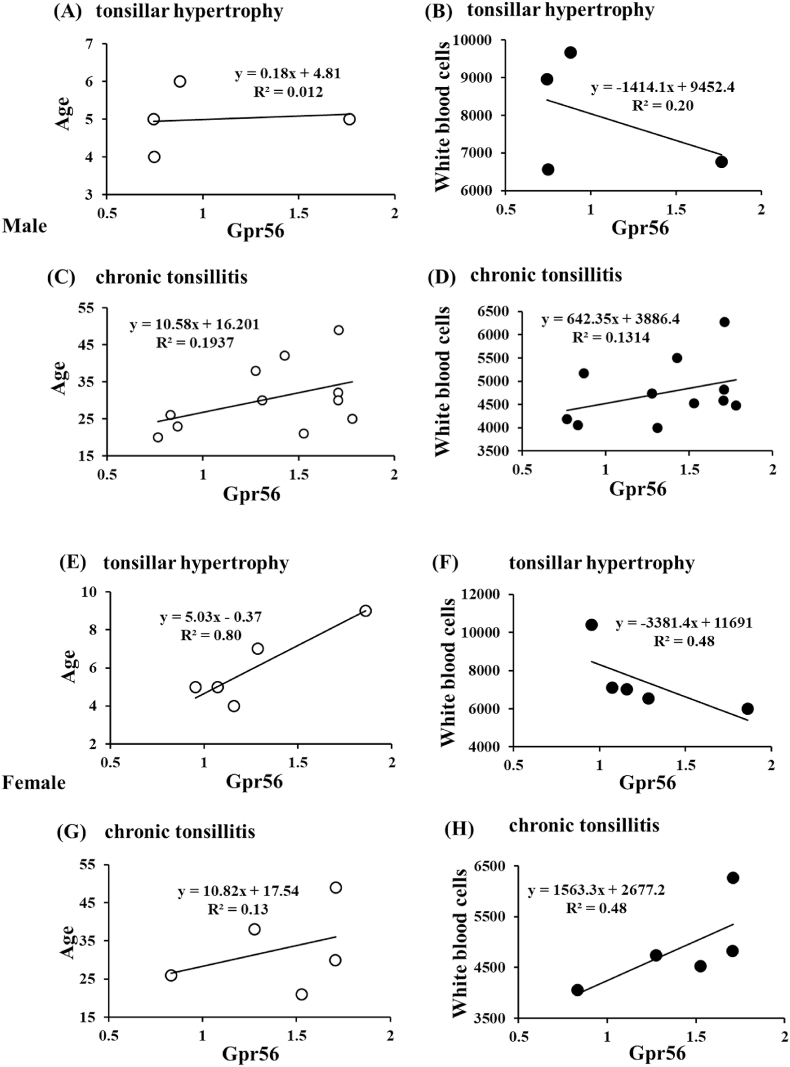
The Gpr56-and sex-dependent age or circulating white blood cell numbers of patients with tonsillar hypertrophy and chronic tonsillitis. The personal data about Gpr56 and age or circulating white blood cell numbers of patients with tonsillar hypertrophy and chronic tonsillitis were separately plot by sex, and the approximate straight line and R^2^ were automatically drawn by Microsoft Excel.

To better study a mechanism of the age-dependent expansion of white blood cells in the development of chronic tonsillitis, patients with tonsillar hypertrophy and chronic tonsillitis were separated by sex and the relationship between Gpr56 and age or white blood cell numbers were analyzed. Gpr56 transcripts were semi-quantitate measured by RT-PCR in palatine tonsils with tonsillar hypertrophy and chronic tonsillitis. There was a positive relationship (Y = 0.18x + 4.8, R^2^ = 0.012) between Gpr56 and age of male patients with tonsillar hypertrophy (Fig. 3A), while there was an inverse relationship (Y = −1414.1x + 9452.4, R^2^ = 0.20) between Gpr56 and white blood cell numbers of male patients with tonsillar hypertrophy (Fig. 3B). Conversely, there was a positive relationship (Y = 10.58x + 16.201, R^2^ = 0.1937) between Gpr56 and age of male patients with chronic tonsillitis (Fig. 3C), and there was a positive relationship (Y = 642.35x + 3886.4, R^2^ = 0.1314) between Gpr56 and white blood cell numbers of male patients with chronic tonsillitis (Fig. 3D). On the other hand, there was a positive relationship (Y = 5.03x – 0.37, R^2^ = 0.80) between Gpr56 and age of female patients with tonsillar hypertrophy (Fig. 3E), while there was an inverse relationship (Y = −3381.4x + 11691, R^2^ = 0.48) between Gpr56 and white blood cell numbers of female patients with tonsillar hypertrophy (Fig. 3F). Conversely, there was a positive relationship (Y = 10.82x + 17.54, R^2^ = 0.13) between Gpr56 and age of female patients with chronic tonsillitis (Fig. 3G), while there was a positive relationship (Y = 1563.3x + 2677.2, R^2^ = 0.48) between Gpr56 and white blood cell numbers of female patients with chronic tonsillitis (Fig. 3H). The R^2^ between Gpr56 and age in male patients was higher than that in female patients with chronic tonsillitis. Importantly, the R^2^ between Gpr56 and white blood cell numbers in female patients was higher than that in male patients with chronic tonsillitis.

### 3.3 The roles of GPR56 in the elder immune system at palate tonsils of female patients with chronic tonsillitis

**Fig. 4:**
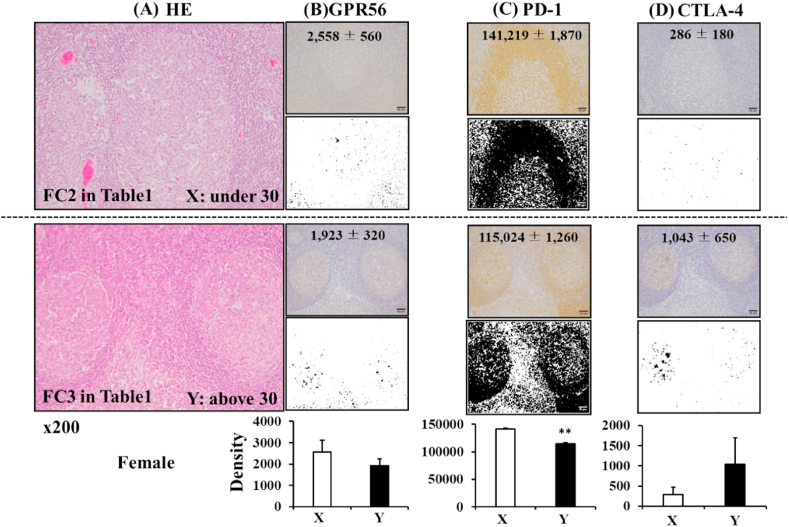
GPR56, PD-1 and CTLA-4 expression at the palatine tonsils of female patients with chronic tonsils in the under and above 30 years old. After paraffin sections at the palatine tonsils of female patients with chronic tonsils in the under (top) and above (bottom) 30 years old were stained with (A) hematoxylin and eosin solution, (B) GPR56, (C) PD-1 and (D) CTLA-4 protein expression were visualized by DBA-H2O2-HRP voltammetry enzyme-linked immunoassay system. The DBA signal levels at magnification 200 times were substantively measured with at least 5 lymphatic follicles and calculated the average and standard deviation by density with NIH ImageJ software.

To examine the GPR56 protein expression at palatine tonsils of female patients with chronic tonsillitis, paraffin sections were stained with hematoxylin and eosin solution (Fig. 4A) or anti-GPR56, anti-PD-1 and anti-CTLA-4 antibodies. The protein localization was pathologically visualized at palatine tonsils by DBA–H_2_O_2_–HRP voltammetry enzyme-linked immunoassay system. The DBA signal levels at magnification 200 times were substantively measured with at least 5 lymphatic follicles and the average and standard deviation by density was calculated with NIH ImageJ software. In our analytic case, when Gpr56 transcript production was not significantly but relatively low at the palatine tonsil extracts of female patients with chronic tonsils, this was considered to be the under 30 years old (FC1-2 in Table 1). The subtractive densities of the visualized GPR56 expression were measured to be 2600 ± 560 mainly at the marginal zone around the lymphatic follicle in the under 30 years old (Fig. 4B top). In the under 30 years old the subtractive densities of the visualized PD-1 expression (140,000 ± 1900) were mainly observed at the marginal zone around the lymphatic follicle (Fig. 4C top). The subtractive densities of the other visualized checkpoint protein CTLA-4 expression were very low (290 ± 180) (Fig. 4D upper). When Gpr56 transcript production was not significantly but relatively high at the palatine tonsil extracts of female patients with chronic tonsils, this was considered to be the above 30 years old (FC3-5 in Table 1).

The subtractive densities of the visualized GPR56 expression were measured to be 1900 ± 320 and observed mainly at the marginal zone around the lymphatic follicle (Fig. 4B lower). In the above 30 years old the subtractive densities of the visualized PD-1 expression (120,000 ± 1300) were mainly observed at the marginal zone around the lymphatic follicle (Fig. 4C bottom). The subtractive densities of the visualized CTLA-4 expression (1000 ± 650) were detected not at marginal zone around the lymphatic follicle but mainly at the germinal center of the lymphatic follicle (Fig. 4D bottom). The statistically significant difference was found in the data for PD-1 expression but not for GPR56 and CTLA-4 expression between the under 30 years old and the above 30 years old. However, the PD-1 expression was high not only the above 30 years old but also the under 30 years old.

**Fig. 5:**
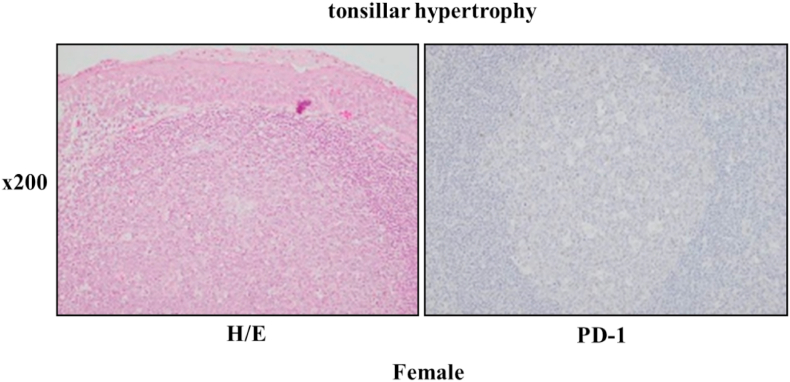
PD-1 expression at the palatine tonsils of female patients with tonsillar hypertrophy. After paraffin sections at the palatine tonsils of female patients with tonsillar hypertrophy were stained with hematoxylin and eosin solution, PD-1 protein expression was visualized by a DBA-H2O2-HRP voltammetry enzyme-linked immunoassay system.

In our analytic case, PD-1 expression was not detected at palatine tonsil of the young patients with tonsillar hypertrophy, this was considered to be the under 10 years old (Fig. 5 and Table 1).

### 3.4 The relationship between PD-1 and Gpr56 expression in chronic tonsillitis in the elder immune system at palate tonsils

**Fig. 6:**
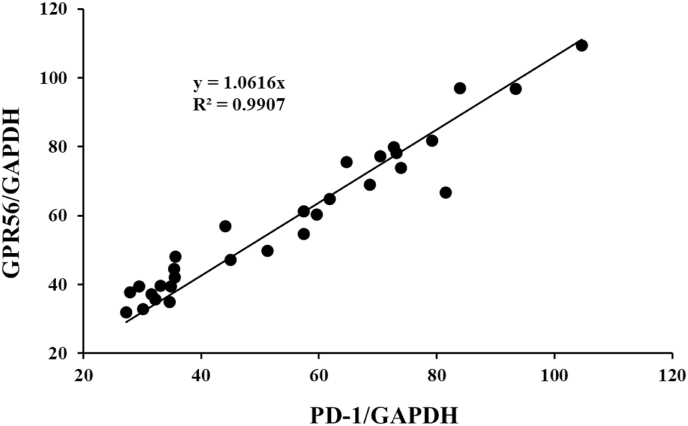
Gpr56 and PD-1 transcripts at the palatine tonsils of the elder patients with chronic tonsils. The personal data about Gpr56 and PD-1 transcripts patients with chronic tonsillitis were separately plot, and the approximate straight line and R^2^ were automatically drawn by Microsoft Excel.

To confirm the role of GPR56 in the proliferation of PD-1^+^ lymphocytes in chronic tonsillitis, palate tonsils of further the elder 39 patients with chronic tonsillitis were collected and analyzed PD-1 and Gpr56 transcripts by the same semi-quantitate RT-PCR method without separation of sex. There was a positive relationship (Y = 1.062x, R^2^ = 0.92) between ratios of PD-1/GAPDH transcripts and ratios of Gpr56/GAPDH transcripts (Fig. 6).

The above data suggested that Gpr56 participated in the age-dependent proliferation of PD-1^+^ lymphocytes in palate tonsils of the elder patients with chronic tonsillitis.

## 4 Discussion

There were transcription factors IRF9 downstream of type I IFNs and NFATc1 downstream of TCR in the promoter region of PD-1 [[9], [10], [11]]. Type I IFNs mediate major innate immune responses to any antigens such as viruses and other infectious agents. The Janus kinase 1 (JAK1) and tyrosine-protein kinase 2 of type I IFN receptors phosphorylate signal transducers and activator of transcription 1 (STAT1) and STAT2. The STAT1/2 heterodimers bind to IRF9 to form IFN-stimulated gene factor 3, which binds to IFN-stimulated response elements in the promoters of IFN-induced genes to initiate their transcription. It is likely that PD-1 expression is regulated via IFN receptors and TCR by both innate and acquired immune responses to antigens.

PD-1 expression was first detected on memory-type CD4^+^ lymphocytes in aged normal mice [12]. Co-activation signal via CD28 with TCR on CD4^+^ lymphocytes is blocked by the PD-1-mediated activation of phosphatase Ptpn11. Therefore, the PD-1^+^/CD4^+^ lymphocytes go into senescence at the marginal zones of the lymphatic follicles. These PD-1^+^/CD4^+^ lymphocytes are usually called long-term CD4^+^ lymphocytes. In this report, there were age ranges of 5.5 ± 1.6 in 9 patients with tonsillar hypertrophy and that of 27.9 ± 10.5 in 13 patients with chronic tonsillitis (Table 1). There were few numbers of long-term CD4^+^ lymphocytes at palatine tonsils with tonsillar hypertrophy (Fig. 5). Conversely, it was found that the main localization of the long-term CD4^+^ lymphocytes was at the marginal zone of the lymphatic follicle. These data indicated that the long-term CD4^+^ lymphocytes activated at palatine tonsils with chronic tonsillitis in the autocrine and/or paracrine manners [13]. However, the effects of the PD-1^+^/CD4^+^ lymphocytes on the local immune system of PD-1^-^/CD4^+^ lymphocytes in the elder patients with chronic tonsillitis is not clearly understood.

There was a report of long-term CD4^+^ lymphocytes in circulating blood in patients with chronic tonsillitis in the above 30 years old [6]. There is no clear answer whether the long-term CD4^+^ lymphocytes are expanded at palate tonsils clonally or in circulation polyclonally. CRPs in patients with chronic tonsillitis were higher than those with tonsillar hypertrophy (Table 1). However, CRP was 0.19 ± 0.30 in patients with chronic tonsillitis. Therefore, PD-1 inducible molecules such as IFN are not continuously released in the blood stream. It is suggested that the long-term CD4^+^ lymphocytes proliferated and moved from the marginal zone around the lymphatic follicle to neighbor vessels. There was one possibility that the JAK-STAT pathway beside the interleukin (IL)-2 receptor makes homodimer of STAT5 resulting in the maintenance of IL-2 receptor expression in an autocrine manner [14]. Cytokines of the common gamma-chain family, including IL-2, IL-7, and IL-15, are critical to both the PD-1 expression and CD4^+^ lymphocyte proliferation [15]. It is likely that the long-term CD4^+^ lymphocytes proliferate at palatine tonsils with chronic tonsillitis in both autocrine and paracrine manners [16]. There was another possibility that memory-type CD4^+^ lymphocytes play a central role in protective immunity against pathogens [8]. Memory-type GPR56^+^/CD4^+^ lymphocyte subsets were found and showed to have higher levels of clonal expansion. The long-term memory GPR56^+^/CD4^+^ lymphocyte subsets likely expanded at the marginal zone of the lymphatic follicles of palatine tonsils with chronic tonsillitis. The authors are very interested in the role of GPR56 in the proliferation of PD-1^+^ cells at the marginal zone of the lymphatic follicles of palatine tonsils of the above 30 years old patients with chronic tonsillitis (Fig. 4). We do not still understand an alternative mechanism of the PD-1^+^ cell proliferation those of the under 30 years old patients. At least, the positive relationship was confirmed between PD-1 transcripts and Gpr56 transcripts (Fig. 6). Moreover, further examination is necessary to understand the effects of the PD-1^+^/CD4^+^ lymphocytes on the elder immune system of PD-1^-^/CD4^+^ lymphocytes in patients with chronic tonsillitis.

CD28 on CD4^+^ lymphocytes interacts with CD80/86 on antigen presenting cells and introduces acute and/or chronic inflammatory cues to antigen-specific B cells at the germinal central zone of the lymphatic follicles for differentiation of plasma cells even in palatine tonsils [17,18]. CTLA-4 expression on T cell subsets commonly thinks to block antigen-specific immunoglobulins by a blockage of the CD28 interaction. Therefore, a delay system against wound healing can sometimes be seen as one of the development mechanisms of chronic inflammation. In this paper, an expression of CTLA-4 at the germinal center of the lymphatic follicles of palatine tonsils was observed in the elder patients with chronic tonsillitis (Fig. 4). The roles of CTLA-4^+^ cells were also observed at chronic inflammation [19].

Chronic inflammation at the synovium in rheumatoid arthritis patients is caused by a predominant accumulation of monocytes/macrophages [20,21]. A monocyte-specific chemoattractant ribosomal protein S19 (RP S19) polymer in rheumatoid arthritis synovial tissues was demonstrated as an alternative ligand of C5a receptor (C5aR), which belongs to the chemotactic G protein-coupled receptor (GPCR) family [22]. It was recently shown that apoptotic cells expressed C5aR and RP S19 monomers which were inter-molecularly cross-linked at 122lysine (Lys: K) and 137Glutamine (Gln: Q) by tissue transglutaminases, whose activation is increased during programmed cell death [23]. To validate the roles of the RP S19 polymer-induced apoptosis promotion system in wound healing of acute inflammation *in vivo*, Q(CAG)137E(GAG) mutant RP S19 gene knock-in C57BL/6J mice (knock-in mice) were prepared [24]. In our experimental setting, the infiltrating neutrophils into the thoracic cavity were almost completely cleared by macrophages for at least 24 h after injection of carrageenan into the control C57BL/6J mice. The senescence neutrophils were confirmed by a blockage of C5aR-mediated apoptosis promotion signal and inactivation of macrophages by another blockage of C5aR-mediated phagocytosis promotion signal at 24 h in the carrageenan-induced acute pleurisy model knock-in mice. Moreover, there were large numbers of lymphocytes, except for the long-termed neutrophils and inactivated macrophages, at 7 days in the thoracic cavity of the carrageenan-induced acute pleurisy model knock-in mice. Their data gave rise to a question whether lymphoid follicles at the inflammatory foci are made of a large number of polyclonally circulating lymphocytes or if clonal lymphocytes infiltrated a small number of circulating lymphocytes. We need to examine a participation of PD-1^+^ lymphocytes proliferated by the GPR56-mediated downstream signal not only in the carrageenan-induced acute pleurisy model knock-in mice but also in rheumatoid arthritis patients.

## CRediT authorship contribution statement

**Yuki Fujihara:** Methodology, Validation, Resources. **Yasuyuki Nagasawa:** Visualization. **Hiroyuki Futani:** Methodology, Validation, Resources. **Shigeo Fukunishi:** Conceptualization, Formal analysis, Investigation, Data curation, Writing - original draft. **Shinichi Yoshiya:** Conceptualization, Formal analysis, Investigation, Data curation, Writing - original draft. **Hiroshi Nishiura:** Conceptualization, Formal analysis, Investigation, Data curation, Writing - original draft.

## Declaration of competing interest

The authors declare no conflict of interest.
